# Clinical Relevance of Serum Galactose Deficient IgA1 in Patients with IgA Nephropathy

**DOI:** 10.3390/jcm9113549

**Published:** 2020-11-04

**Authors:** Jin Sug Kim, Hyeon Seok Hwang, Sang Ho Lee, Yang Gyun Kim, Ju-Young Moon, Ji Yoon Kong, Kyung Hwan Jeong

**Affiliations:** 1 Division of Nephrology, Department of Internal Medicine, College of Medicine, Kyung Hee University, Seoul 02453, Korea; jinsuk0902@naver.com (J.S.K.); hwanghsne@gmail.com (H.S.H.); lshkidney@khu.ac.kr (S.H.L.); apple8840@hanmail.net (Y.G.K.); kidmjy@hanmail.net (J.-Y.M.); 2 Department of Medicine, Graduate School, Kyung Hee University, Seoul 02453, Korea; faintgreen@naver.com

**Keywords:** IgA nephropathy, galactose deficient IgA1, biomarker

## Abstract

New biomarkers of IgA nephropathy (IgAN) are needed for non-invasive diagnosis and appropriate treatment. There is emerging evidence that galactose deficient IgA1 (Gd-IgA1) is a pivotal molecule in the pathogenesis of IgAN. However, few studies have investigated the role of Gd-IgA1 as a biomarker in IgAN. In this study, we investigated the clinical relevance of serum Gd-IgA1 levels in patients with IgAN. Two hundred and thirty biopsy-proven IgAN patients, 74 disease controls (patients with non-IgAN nephropathy), and 15 healthy controls were enrolled in this study. Levels of serum Gd-IgA1 were measured using an ELISA kit in serum samples obtained the day of renal biopsy. We compared levels of serum Gd-IgA1 according to the type of glomerular disease and analyzed the association between Gd-IgA1 levels and clinical and pathological parameters in patients with IgAN. We then divided IgAN patients into two groups according to Gd-IgA1 level and investigated the predictive value of Gd-IgA1 for progression of chronic kidney disease (CKD). Serum Gd-IgA1 levels were significantly higher in IgAN patients than disease controls and healthy controls. In patients with IgAN, serum Gd-IA1 levels were significantly correlated with estimated glomerular filtration rate, serum IgA level, and tubular atrophy/interstitial fibrosis. CKD progression was more frequent in IgAN patients with higher serum Gd-IgA1 levels than in those with lower serum Gd-IgA1 levels. Cox proportional hazard models showed that high GdIgA1 level was an independent risk factor for CKD progression after adjusting for several confounders. Our results suggest that serum Gd-IgA1 level is a useful diagnostic and prognostic marker in IgAN patients. Further studies with a larger sample size and longer follow-up duration are needed.

## 1. Introduction

IgA nephropathy (IgAN) is the most frequent form of primary glomerulonephritis and one of the important causes of chronic kidney disease (CKD) worldwide [1]. The clinical course and disease prognosis of IgAN patients vary, and about 20–40% of patients reach end stage renal disease (ESRD) within 20 years of diagnosis [2,3]. Therefore, early diagnosis, risk prediction for disease progression, and appropriate treatment are important in IgAN. However, the pathogenesis of this disease is not yet fully understood and curative treatment strategies remain to be established [4]. Although the current gold standard diagnostic and prognostic method for IgAN is renal biopsy, it is not frequently performed in the real clinical field due to some limitations and concerns about complications [4,5]. Thus, it is necessary to identify non-invasive biomarkers that can be used to diagnose IgAN and assess activity and outcomes of the disease.

Several studies have shown that aberrant IgA1 O-linked glycosylation plays a key role in the pathogenesis of IgAN [6,7]. Moldoveanu et al. [8] demonstrated that serum levels of galactose-deficient IgA1 (Gd-IgA1) were increased in patients with IgAN. The multi-hit hypothesis of the pathogenesis of IgAN states that antibodies directed against overproduced Gd-IgA1 are generated and formed immune complexes. These immune complexes subsequently accumulate in the glomerular mesangium and cause kidney injury [9]. Gd-IgA1 was suggested as a potential disease-specific biomarker that predicts disease activity and prognosis. Some studies reported serum Gd-IgA1 as a diagnostic marker for IgAN [8,10]. Other researchers showed that high serum levels of Gd-IgA1 are associated with disease severity and progression [11,12]. However, further studies are needed because most of the previous studies have involved a relatively small sample size and results have been inconsistent.

In this study, we measured levels of serum Gd-IgA1 in over 200 patients with IgAN and compared them with those in controls. We then investigated the associations between serum Gd-IgA1 levels and clinicopathological parameters in IgAN patients. The predictive value of serum Gd-IgA1 for CKD progression was also analyzed based on more than 3 years of follow-up observations.

## 2. Materials and Methods

### 2.1. Study Population and Design

We enrolled 230 patients with biopsy-proven IgAN from two hospitals (Kyung Hee University Medical Center and Kyung Hee University Hospital at Gangdong) in Seoul, Korea, from January 2007 to November 2017. We also enrolled patients with non-IgAN nephropathy as disease controls: 35 patients with membranous nephropathy (MN), 21 patients with minimal change disease (MCD), eight with lupus nephritis (LN), and 10 with thin basement membrane disease (TBMD). Fifteen subjects without kidney disease were included as healthy controls. We compared clinical characteristics, laboratory findings, and serum levels of Gd-IgA1 between IgAN patients and controls. We then investigated associations of Gd-IgA1 levels with clinical and pathological parameters in IgAN patients. IgAN patients were divided into two groups according to median level of serum Gd-IgA1 (lower Gd-IgA1 and higher Gd-IgA1 groups) to analyze the associations between Gd-IgA1 level and clinical outcome.

All study procedures complied with the ethical guidelines of the Declaration of Helsinki and were approved by the Institutional Review Board of each hospital. The approval number from Kyung Hee University Medical Center is 2009-06-301. Written consent was obtained from all participants.

### 2.2. Clinical and Pathological Parameters

Baseline variables including age, sex, body mass index (BMI), and medications that had been taken before renal biopsy were recorded. Blood samples were obtained for measurement of serum albumin, creatinine, and IgA, and urine was collected to assess the amount of proteinuria and presence of hematuria at the time of renal biopsy. The amount of proteinuria was calculated as the spot urine protein to creatinine ratio (PCR). Renal function was assessed via the estimated glomerular filtration rate (eGFR), calculated using the Chronic Kidney Disease Epidemiology Collaboration (CKD-EPI) equation [13]. IgAN pathology was described using the Oxford classification system [14].

### 2.3. Measurement of Serum Gd-IgA1

Serum samples were collected on the day of biopsy and stored at −70 °C. Level of serum Gd-IgA1 was measured using a commercially available enzyme-linked immunosorbent assay test kit with KM55 (IBL, Fujioka, Japan) according to the manufacturer’s protocol.

### 2.4. Treatment and Clinical Outcome

Patients diagnosed with IgAN were treated with an angiotensin receptor blocker (ARB) or angiotensin-converting enzyme inhibitor (ACEi) alone or in combination with immunosuppressants. Patients who were taking an ARB or ACEi prior to renal biopsy continued to take an ARB or ACEi after the biopsy. Patients visited the outpatient clinic regularly every 1–2 months for the assessment of renal function and proteinuria. Clinical outcome of this study was CKD progression, defined as a greater than 25% reduction in eGFR or decline in eGFR category from the value determined at the time of renal biopsy [15].

### 2.5. Statistical Analysis

All statistical analyses were conducted using SPSS software version 19.0 (SPSS Inc., Chicago, IL, USA) and R software version 4.0.2 (R Foundation for Statistical Computing, Vienna, Austria). Continuous variables are presented as the median (first quartile-third quartile) and categorical data are reported as the frequency and percentage. Comparisons of continuous variables were performed by independent t test or Mann-Whitney U test as appropriate. Categorical variables were compared using Chi-square test. The differences of clinical parameters among glomerular disease groups were evaluated by a Kruskal-Wallis test followed by a multiple comparison analysis. The Bonferroni test was used as it was appropriate for post hoc analysis. To find the optimal cut-off value of Gd-IgA1, the hazard ratio for all cases of Gd-IgA1 values was calculated. In addition, we selected the Gd-IgA1 value, which represents the maxed hazard ratio (=highest accuracy). Correlations among variables were assessed using Spearman’s rank correlation coefficient test. Survival curves were estimated by the Kaplan-Meier method and compared with the log-rank test. Univariate and multivariate Cox regression analyses were used to investigate the independent association of Gd-IgA1 level with CKD progression. Variables with a *p*-value < 0.10 in the univariate Cox regression analyses were selected for multivariate Cox regression analysis. Results are presented as hazard ratios (HRs) ±95% confidence intervals (CIs), and statistical significance is indicated. A *p*-value < 0.05 was considered statistically significant.

## 3. Results

### 3.1. Baseline Characteristics of Study Population

**Figure 1:**
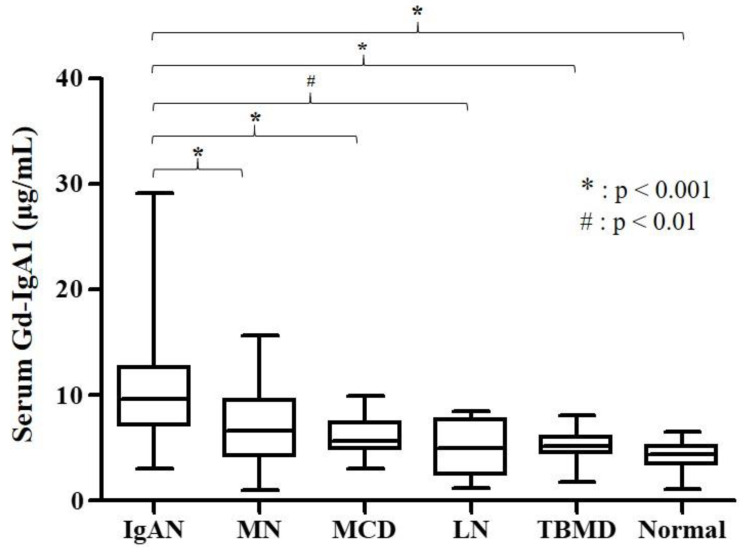
Serum Gd-IgA1 levels according to type of glomerular disease. IgAN, IgA nephropathy; MN, membranous nephropathy; MCD, minimal change disease; LN, lupus nephritis; TBMD, thin basement membrane disease; Gd-IgA1, galactose-deficient IgA1. *: *p* < 0.001 and #: *p* < 0.01. Multiple comparisons for the level of serum Gd-IgA1 was performed by a Bonferroni significant difference test. Box plot shows median, first and third quartiles, minimum and maximum.

The baseline characteristics of the participants in the IgAN group, disease control group, and healthy control group are summarized in Table 1. The median age of the IgAN patients was 41.00 years, and 50% were male. IgAN patients showed significantly higher levels of serum albumin than patients with MN, MCD, and LN, and lower levels of serum albumin than patients with TBMD and healthy controls. Patients with MN and MCD excreted significantly more urinary protein than IgAN patients. Levels of serum Gd-IgA1 were significantly elevated in patients with IgAN compared to disease controls and healthy controls (Table 1 and Figure 1).

### 3.2. Association of Serum Gd-IgA1 Level with Clinical and Pathological Parameters in IgAN Patients

**Figure 2:**
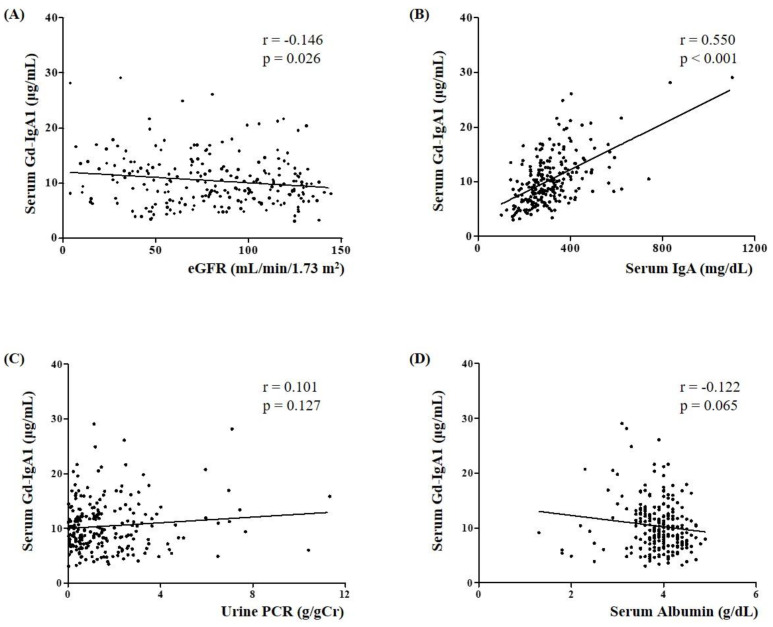
Relationship between serum Gd-IgA1 level and clinicopathological characteristics (**A**: eGFR; **B**: serum IgA; **C**: urine PCR; and **D**: serum albumin) in patients with IgAN. Gd-IgA1, galactose-deficient IgA; IgAN, IgA nephropathy; eGFR, estimated glomerular filtration rate; PCR, protein to creatinine ratio. Data were statistically analyzed using Spearman’s rank correlation coefficient test.

Figure 2 shows the correlation between serum Gd-IgA1 level and clinical parameters in 230 IgAN patients. The Gd-IgA1 level showed a weak negative correlation with eGFR (r = −0.146, *p* = 0.026, Figure 2A) and a positive correlation with serum IgA level (r = 0.550, *p* < 0.001, Figure 2B). However, neither urine PCR nor albumin was significantly correlated with Gd-IgA1 level (*p* = 0.127 and *p* = 0.065, respectively) (Figure 2C,D). Based on the Oxford classification, serum Gd-IgA1 level was significantly elevated in patients with tubular atrophy/interstitial fibrosis (T0, 9.15 (6.93–12.18) vs. T1-2, 10.93 (8.45–16.69), *p* = 0.024) (Table 2).

### 3.3. Association of Serum Gd-IgA1 Level and CKD Progression in IgAN Patients

**Figure 3:**
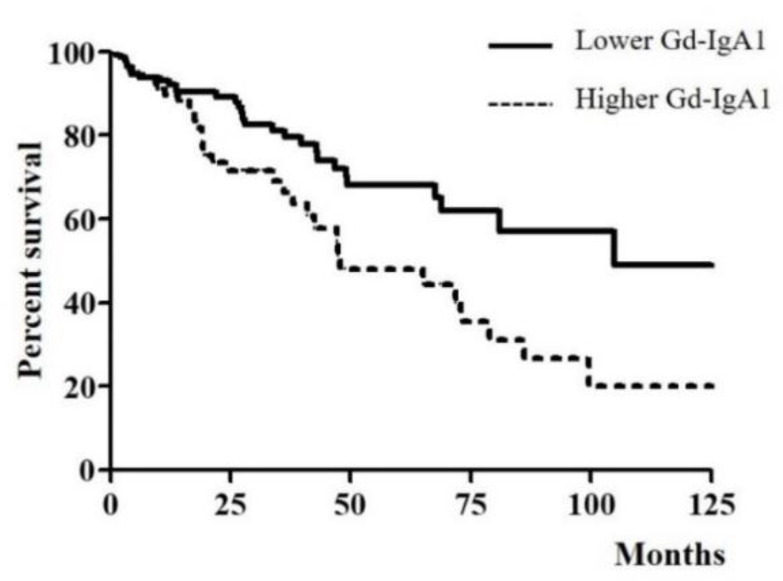
Kaplan-Meier curve for CKD progression-free survival of IgAN patients according to serum Gd-IgA1 levels.

IgAN patients were divided into two groups according to the optimal cut-off value of serum Gd-IgA1 which obtained by the aforementioned method: a lower Gd-IgA1 group (Gd-IgA1 < 11.31 μg/mL, *n* = 148) and a higher Gd-IgA1 group (Gd-IgA1 ≥ 11.31 μg/mL, *n* = 82). The characteristics of the two groups are compared in Table 3. Patients in the higher Gd-IgA1 group had a significantly lower serum albumin level and eGFR (*p* = 0.012 and *p* = 0.001, respectively), and higher serum IgA level (*p* < 0.001) than those in the lower Gd-IgA1 group. No significant differences were observed between the two groups with regard to age, sex, BMI, urine PCR, urine RBC grade, or drug usage. Median follow-up duration was 22.55 months and 64 patients (27.8%) experienced CKD progression. More patients in the higher Gd-IgA1 group showed CKD progression than those in the lower Gd-IgA1 group (40.2% in the higher Gd-IgA1 group versus 20.9% in the lower Gd-IgA1 group, *p* = 0.002). Figure 3 shows CKD progression according to serum Gd-IgA1 level. A log-rank test identified a significant association between serum Gd-IgA1 level and CKD progression (*p* = 0.006).

We conducted univariate and multivariate Cox regression analyses to identify risk factors associated with CKD progression in IgAN patients (Table 4). In univariate Cos regression analysis, age (HR 1.029, 95% CI 1.012–1.046, *p* = 0.001), eGFR (HR 0.985, 95% CI, 0.978–0.991, *p* < 0.001), urine PCR (HR 1.191, 95% CI, 1.081–1.313, *p* < 0.001), and higher serum Gd-IgA1 (HR 2.283, 95% CI, 1.388–3.756, *p* = 0.01) showed a significant association with CKD progression in IgAN patients. After adjustment for variables with a *p*-value < 0.10 in the univariate analysis, eGFR (HR 0.991, 95% CI, 0.982–0.999, *p* = 0.048) and higher serum Gd-IgA1 (HR 1.933, 95% CI, 1.164–3.208, *p* = 0.011) were independent factors associated with CKD progression in IgAN patients.

## 4. Discussion

In this study, we measured serum Gd-IgA1 level and investigated its clinical relevance in patients with IgAN. Our major findings were (1) serum Gd-IgA1 levels in patients with IgAN were significantly higher than those in disease controls and healthy controls; (2) serum Gd-IgA1 level was negatively correlated with eGFR and positively correlated with serum IgA in patients with IgAN; (3) serum Gd-IgA1 level was significantly elevated in IgAN patients with tubular atrophy/interstitial fibrosis; (4) CKD progression was more frequent in IgAN patients with a higher level of serum Gd-IgA1 than those with a lower serum Gd-IgA1 level; (5) higher serum Gd-IgA1 was an independent predictor of CKD progression in patients with IgAN.

Although there have been remarkable advances since IgAN was first described by Berger et al. in 1968 [16], the pathogenesis of the disease is not yet fully understood and there are currently no disease-specific biomarkers that are reliable and useful in clinical practice [4,11]. Despite the proposal of several candidate biomarkers in recent years, these biomarkers lack sensitivity and specificity [5]. Pathologic findings and nonspecific clinical parameters such as eGFR, urine protein excretion, and blood pressure are therefore currently used to assess disease activity and prognosis in IgAN [17,18].

The multi-hit hypothesis of IgAN pathogenesis is widely accepted. This multi-hit hypothesis proposes the following disease pathogenesis: first, an increase in aberrant glycosylation of IgA1 leading to overproduction of Gd-IgA1; second, synthesis of antibodies that recognize Gd-IgA1; third, formation of pathogenic immune complexes; and fourth, mesangial deposition of these complexes and initiation of renal injury [4,9]. Several studies have provided evidence supporting the multi-hit hypothesis, and Gd-IgA1 is therefore drawing attention as a potential biomarker of IgAN [19].

Previous studies have revealed that IgAN patients have significantly higher levels of serum Gd-IgA1 than patients with non-IgAN glomerular diseases and healthy subjects [8,12]. Consistent with prior studies, we observed a significantly increased serum Gd-IgA1 level in IgAN patients compared with disease controls and healthy controls. Considering that Gd-IgA1 level could be affected by serum IgA level, we further analyzed the serum Gd-IgA1 to IgA ratio (Supplementary Table S1). Serum Gd-IgA1 to IgA ratio was significantly elevated in patients with IgAN as compared with in patients with MN, MCD, and LN, and healthy controls. TBMD patients had lower Gd-IgA1 to IgA ratio than IgAN patients, but there was no statistical significance. Further studies are needed to validate our findings and confirm the clinical relevance of the Gd-IgA1 to IgA ratio.

Several study groups have demonstrated that Gd-IgA1 has clinical significance in patients with IgAN. Zhao et al. [20] showed that elevated serum Gd-IgA1 levels were associated with aggravation of urinary protein excretion and increased risk of renal function deterioration in IgAN patients. Other studies have also demonstrated that serum Gd-IgA1 levels were correlated with disease severity and renal outcome of IgAN [11,12]. In our study, serum level of Gd-IgA1 was correlated with eGFR, and the frequency of CKD progression was greater in IgAN patients with a higher serum Gd-IgA1 level than those with a lower serum Gd-IgA1 level. Multivariate Cox regression analysis revealed that higher serum Gd-IgA1 level was an independent risk factor for CKD progression.

Associations between serum Gd-IgA1 level and pathologic findings have also been reported. Xu et al. [21] showed that serum Gd-IgA1 level was closely associated with pathologic phenotypes in IgAN. They compared serum IgA1 glycosylation between IgAN patients with different pathologic phenotypes and observed that levels of α2,6 sialic acid and galactose of serum IgA1 were significantly lower in patients with focal proliferative sclerosing IgAN than those with in mild mesangial proliferative IgAN. Wada et al. [11] reported that serum Gd-IgA1 levels were significantly higher in IgAN patients with segmental sclerosis and tubular atrophy/interstitial fibrosis than those who did not have this condition. Consistent with this study, we also observed that serum Gd-IgA1 level was associated with tubular atrophy/interstitial fibrosis in IgAN patients. To our knowledge, the mechanism for the association between serum Gd-IgA1 levels and tubulointerstitial lesion has not been studied previously. Zhang et al. [10] showed the relationship between serum Gd-IgA1 levels and deposition of mesangial Gd-IgA1. Mesangial cells injured by deposition of Gd-IgA1 are reported to promote glomerulotubular cross-talk by secreting mediators such as cytokines and complements [22,23]. We plan to conduct further study assuming that this process might be related to the association between serum Gd-IgA1 level and tubular atrophy/interstitial fibrosis in IgAN patients. Further studies are needed to elucidate the underlying mechanisms.

A snail helix aspersa agglutinin (HAA) lectin-based assay is currently used to measure serum Gd-IgA1 level. Although the HAA lectin-based assay is a useful research tool and is widely used, the assay is complex to perform and there are issues with the bioactivity and stability of purified lectin [10,12]. Recently, a Gd-IgA1-specific antibody named KM55 was developed for lectin-independent assays [24]. Suzuki et al. demonstrated that KM55 recognizes Gd-IgA1 in IgAN patients as well as the HAA lectin-based assay does [24,25], and some studies have measured Gd-IgA1 using this assay and investigated the clinical significance of Gd-IgA1 in patients with IgAN [10,12]. In this study, we measured serum Gd-IgA1 levels using a lectin-independent assay and observed the similar trends to those reported in previous studies.

Our study had some potential limitations. First, the level of serum Gd-IgA1 was measured only once, which may have resulted in an incorrect classification of patients. To overcome this limitation, we are planning to build an additional independent cohort and test the diagnostic value of Gd-IgA1 as a biomarker. Using the cohort, we also plan to monitor Gd-IgA1 levels during the follow-up period and determine the prognostic value of Gd-IgA1 and its ability to assess the therapeutic effect. Second, urine protein level was measured in a spot urine sample. Third, the cut-off value of higher and lower serum Gd-IgA1 was suggested with appropriate calculation, but further studies with large sample sizes are needed to ascertain the reliability of the cut-off value. Despite these limitations, we demonstrated the clinical relevance of Gd-IgA1 by performing a long-term follow-up (mean follow-up period over 3 years) in a relatively large number of IgAN patients.

In summary, we found that serum Gd-IgA1 levels were noticeably elevated in patients with IgAN and were significant associated with clinicopathological variables. Higher serum Gd-IgA1 level predicted CKD progression in IgAN patients. Serum Gd-IgA1 is therefore a potential disease-specific biomarker for diagnosis and assessment of the disease progression of IgAN. Further studies with a larger sample size and longer follow-up are needed to confirm our findings.

## Figures and Tables

**Table 1:** Baseline characteristics of the study population according to the type of glomerular disease.

	IgAN (*n* = 230)	MN (*n* = 35)	MCD (*n* = 21)	LN (*n* = 8)	TBMD (*n* = 10)	Heathy Controls (*n* = 15)
Age (years)	41.00 (31.00–52.00) ^b^	53.00 (41.25–63.75) ^a^	48.00 (21.00–62.00)	44.00 (22.00–53.00)	40.00 (20.50–46.50) ^b^	25.00 (21.75–50.50) ^b^
Male (*n*, %)	115 (50.0%)	23 (65.7%)	13 (61.9%)	0 (0.0%)	5 (50.0%)	12 (80.0%)
BMI (kg/m^2^)	23.50 (21.28–25.68)	23.19 (22.12–24.28)	25.20 (21.79–28.25)	21.75 (19.90–22.47)	23.29 (21.75–24.77)	22.32 (20.95–24.23)
Albumin (g/dL)	3.90 (3.60–4.20) ^b,c,d,f^	3.35 (2.57–4.10) ^a,c,e,f^	2.20 (1.90–2.40) ^a,b,e,f^	2.90(2.22–3.60) ^a,e,f^	4.50(4.15–4.65) ^b,c,d^	4.60 (4.33–4.73) ^a,b,c,d^
Creatinine (mg/dL)	0.94 (0.74–1.38)	0.80 (0.60–1.03)	0.90 (0.70–1.45)	0.60 (0.56–1.87)	0.73 (0.55–0.85)	0.76 (0.67–0.99)
eGFR (mL/min/1.73 m^2^)	84.18 (52.50–113.91) ^e^	90.90 (77.91–115.59)	85.32 (53.20–112.16)	110.89 (31.06–132.90)	121.75 (110.69–130.39) ^a^	125.29 (87.29–137.78)
C3 (mg/dL)	107.00 (92.00–121.00) ^d^	112.00 (95.52–125.25) ^d^	114.00 (105.50–129.00) ^d^	45.95 (32.83–56.75) ^a,b,c,e,f^	97.40 (89.45–105.00) ^d^	102.00 (84.07–111.25) ^d^
IgA (mg/dL)	287.0 (240.00–361.50) ^e,f^	214.00 (175.50–283.75)	264.00 (220.00–353.00)	264.00 (220.00–353.00)	177.00 (151.00–199.50) ^a^	171.00 (124.00–204.50) ^a^
Urine PCR (g/gCr)	1.24 (0.46–2.34) ^b,c^	3.89 (1.64–6.22) ^a,c,e,f^	8.63 (4.81–13.95) ^a,b,d,e,f^	2.28 (0.62–4.09) ^c^	0.60 (0.03–0.63) ^b,c^	0.04 (0.19–0.07) ^b,c^
Urine RBC grade						
<5/HPF	45 (19.6%)	17 (48.6%)	12 (57.1%)	1 (12.5%)	1 (10.0%)	–
5–9/HPF	30 (13.0%)	6 (17.1%)	6 (28.6%)	2 (25.0%)	3 (30.0%)	–
10–29/HPF	59 (25.7%)	6 (17.1%)	1 (4.8%)	2 (25.0%)	5 (50.0%)	–
≥30/HPF	96 (41.7%)	6 (17.1%)	2 (9.5%)	3 (37.5%)	1 (10.0%)	–
Serum Gd–IgA1 (μg/mL)	9.66 (7.14–12.60)^b,c,d,e,f^	6.65 (4.21–9.51) ^a^	5.60 (4.86–7.38) ^a^	4.95 (2.40–7.71) ^a^	5.19 (4.71–6.16) ^a^	4.43 (3.44–5.15) ^a^

IgAN, IgA nephropathy; MN, membranous nephropathy; MCD, minimal change disease; LN, lupus nephritis; TBMD, thin basement membrane disease; BMI, body mass index; eGFR, estimated glomerular filtration rate; PCR, protein to creatinine ratio; Gd-IgA1, galactose-deficient IgA1. ^a^: *p* < 0.05, vs. IgAN; ^b^: *p* < 0.05, vs. MN; ^c^: *p* < 0.05, vs. MCD; ^d^: *p* < 0.05, vs. LN; ^e^: *p* < 0.05, vs. TBMD; ^f^: *p* < 0.05, vs. healthy control. Continuous variables are presented as the median (first quartile-third quartile). The multiple comparisons for continuous variables were performed by a Bonferroni test. Categorical data were determined by a Chi-square test.

**Table 2:** Serum Gd-IgA1 levels according to pathologic findings based on the Oxford classification (MEST-C).

Oxford Classification		*n* (%)	Serum Gd-IgA1 (μg/mL)	*p*
M	0	135 (58.7%)	8.77 (6.64–12.17)	0.414
	1	95 (41.3%)	10.57 (7.94–13.46)	
E	0	174 (76.5%)	9.41 (7.12–13.29)	0.898
	1	56 (23.5%)	9.91 (7.61–12.17)	
S	0	167 (72.6%)	9.73 (7.19–12.47)	0.672
	1	63 (27.4%)	9.42 (7.12–12.95)	
T	0	193 (83.9%)	9.15 (6.93–12.18)	0.024
	1,2	37 (16.1%)	10.93 (8.45–16.69)	
C	0	170 (73.9%)	9.89 (7.19–13.00)	0.268
	1,2	60 (26.1%)	8.69 (6.22–12.16)	

Gd-IgA1, galactose-deficient IgA1; M, mesangial hypercellularity; E, endocapillary hypercellularity; S, segmental glomerulosclerosis; T, tubular atrophy/interstitial fibrosis; C, cellular or fibrocellular crescents. Data were statistically analyzed using Mann-Whitney U test. Continuous variables are presented as the median (first quartile-third quartile).

**Table 3:** Clinical characteristics of IgAN patients according to serum Gd-IgA1 level.

	Lower Gd-IgA1 (<11.31 μg/mL) *n* = 148	Higher Gd-IgA1 (≥11.31 μg/mL) *n* = 82	*p*
Age (years)	40.00 (26.50–52.00)	42.00 (34.00–49.00)	0.238
Male (*n*, %)	74 (50%)	41 (50%)	0.999
BMI (kg/m^2^)	23.50 (21.16–25.71)	23.44 (21.48–25.65)	0.794
Serum albumin (g/dL)	4.00 (3.70–4.30)	3.80 (3.50–4.10)	0.012
Serum creatinine (mg/dL)	0.90 (0.74–1.19)	1.10 (0.80–1.70)	0.001
eGFR (mL/min/1.73 m^2^)	92.96 (59.44–118.15)	72.59 (41.83–108.38)	0.001
C3 (mg/dL)	107.00 (95.05–120.50)	107.0 (92.00–423.00)	0.478
Serum IgA (mg/dL)	269.00 (228.50–324.00)	354.0 (278.00–423.00)	<0.001
Urine PCR (g/gCr)	1.11 (0.36–2.33)	1.22 (0.51–2.44)	0.337
Prior medications (*n*, %)			
ARB or ACEi	33 (22.3%)	22 (26.8%)	0.440
CCB	23 (15.5%)	9 (11.0%)	0.226
Beta blocker	6 (4.1%)	3 (3.7%)	0.593
Statin	10 (6.8%)	5 (6.1%)	0.543
Urine RBC grade (*n*, %)			0.867
<5/HPF	28 (18.9%)	17 (20.7%)	
5–9/HPF	21 (14.2%)	9 (11.0%)	
10–29/HPF	39 (26.4%)	20 (24.4%)	
≥30/HPF	60; (40.5%)	36 (43.9%)	
Serum Gd–IgA1 (μg/mL)	7.95 (6.23–9.36)	13.84 (12.44–16.73)	<0.001
Therapeutic strategies (*n*, %)			
ARB or ACEi	103 (69.6%)	57 (69.5%)	0.990
Immunosuppressant	28 (55.4%)	46 (56.1%)	0.515
Follow–up duration (months)	22.55 (11.68–45.83)	22.41 (13.05–42.32)	0.998
CKD progression	31 (20.9%)	33 (40.2%)	0.002

BMI, body mass index; eGFR, estimated glomerular filtration rate; PCR, protein to creatinine ratio; Gd-IgA1, galactose-deficient IgA1; ARB, angiotensin receptor blocker; ACEi, angiotensin-converting enzyme inhibitor; CCB, calcium channel blocker; CKD, chronic kidney disease. Continuous variables are presented as the median (first quartile-third quartile). Mann-Whitney U tests were used to compare continuous variables between the groups. A Chi-square test was used to compare the categorical variables between the groups.

**Table 4:** Predictors of CKD progression in univariate and multivariate Cox regression analyses.

Variables	Univariate Analysis		Multivariate Analysis	
	HR (95% CI)	*p*	HR (95% CI)	*p*
Age (years)	1.029 (1.012–1.046)	0.001	1.016 (0.996–1.035)	0.111
Male (vs. Female)	1.076 (0.659–1.759)	0.769		
BMI (kg/m^2^)	0.997 (0.921–1.079)	0.939		
eGFR (mL/min/1.73 m^2^)	0.985 (0.978–0.991)	<0.001	0.991 (0.982–0.999)	0.048
Urine PCR(g/g)	1.191 (1.081–1.313)	<0.001	1.116 (0.991–1.256)	0.070
Prior medications				
ARB or ACEi	1.587 (00.903–2.789)	0.109		
CCB	1.420 (0.698–2.888)	0.333		
Beta blocker	2.540 (0.783–8.268)	0.120		
Statin	1.070 (0.333–3.438)	0.909		
Oxford classification				
M	1.226 (0.747–2.012)	0.420		
S	1.502 (0.885–2.550)	0.132		
E	1.340 (0.733–2.451)	0.341		
T	1.698 (0.955–3.018)	0.071	1.151 (0.614–2.158)	0.661
C	1.532 (0.870–2.699)	0.140		
Therapeutic strategies				
ARB or ACEi	1.523 (0.857–2.706)	0.151		
Immunosuppressant	1.344 (0.796–2.269)	0.268		
Lower serum Gd–IgA1 level	1			
Higher serum Gd–IgA1 level	2.283 (1.388–3.756)	0.001	1.933 (1.164–3.208)	0.011

CKD, chronic kidney disease; BMI, body mass index; eGFR, estimated glomerular filtration rate; PCR, protein to creatinine ratio; ARB, angiotensin receptor blocker; ACEi, angiotensin-converting enzyme inhibitor; CCB, calcium channel blocker; Gd-IgA1, galactose-deficient IgA1. Variables with a *p*-value < 0.10 in the univariate analysis were selected for the multivariate cox analysis.

## Supplementary Materials

The following are available online at https://www.mdpi.com/2077-0383/9/11/3549/s1, Table S1: Serum Gd-IgA1 to IgA ratio according to the type of glomerular disease.
