# Supplementary material for: Clinical Relevance of Serum Galactose Deficient IgA1 in Patients with IgA Nephropathy
Source: J Clin Med. 2020 Nov 4;9(11):3549. doi: 10.3390/jcm9113549 (PMC7694202; doi:10.3390/jcm9113549)
Supplement: Supplementary file 1 [file jcm-09-03549-s001.pdf]

Table S1. Serum Gd-IgA1 to IgA ratio according to the type of glomerular disease.

	**IgAN (*n* = 230)**	**MN (*n* = 35)**	**MCD (*n* = 21)**	**LN (*n* = 8)**	**TBMD (*n* = 10)**	**Heathy Controls (*n* = 15)**
Serum Gd-IgA1/IgA (μg/mg IgA)	3.13 (2.42–4.31) [b,c,d,f]	2.06 (1.76–2.77) [a]	2.09 (1.64–2.54) [a]	1.67 (1.08–1.93) [a]	2.65 (2.38–2.96)	2.42 (2.03–2.51) [a]

IgAN, IgA nephropathy; MN, membranous nephropathy; MCD, minimal change disease; LN, lupus nephritis; TBMD, thin basement membrane disease; Gd-IgA1, galactose-deficient IgA1. [a]: $p < 0.05$, vs. IgAN; [b]: $p < 0.05$, vs. MN; [c]: $p < 0.05$, vs. MCD; [d]: $p < 0.05$, vs. LN; [e]: $p < 0.05$, vs. TBMD; [f]: $p < 0.05$, vs. healthy control. Serum Gd-IgA to IgA ratio is presented as the median (first quartile-third quartile). The multiple comparisons for continuous variables were performed by Bonferroni test.
